# Monitoring Interfacial Dynamics of a Zinc‐Ion Battery Cathode Using In Situ Grazing Incidence X‐Ray Absorption Spectroscopy: A Case Study of Manganese Dioxide

**DOI:** 10.1002/smtd.202500871

**Published:** 2025-08-05

**Authors:** Wathanyu Kao‐ian, Phonnapha Tangthuam, Pinit Kidkhunthod, Wanwisa Limphirat, Jintara Padchasri, Nicolas Aubert, Gianluca Ciatto, Insik In, Kevin C.‐W. Wu, Soorathep Kheawhom

**Affiliations:** ^1^ Department of Chemical Engineering Faculty of Engineering Chulalongkorn University Bangkok 10330 Thailand; ^2^ Synchrotron Light Research Institute (Public Organization) 111 University Avenue, Muang District Nakhon Ratchasima 30000 Thailand; ^3^ Synchrotron SOLEIL L'Orme des Merisiers Départementale 128 Saint‐Aubin 91190 France; ^4^ Department of Polymer Science and Engineering Chemical Industry Institute Korea National University of Transportation Chungju 27469 South Korea; ^5^ Department of IT‐Energy Convergence (BK21 FOUR) Korea National University of Transportation Chungju 27469 South Korea; ^6^ Department of Chemical Engineering National Taiwan University Taipei 10617 Taiwan; ^7^ Department of Chemical Engineering and Materials Science Yuan Ze University Chung‐Li Taoyuan 32003 Taiwan; ^8^ Center of Excellence on Advanced Materials for Energy Storage Chulalongkorn University Bangkok 10330 Thailand

**Keywords:** cathode degradation, energy storage, In situ characterization, interfacial mechanisms, surface reconstruction

## Abstract

Despite such assets as intrinsic safety and low cost, the performance of zinc‐ion batteries (ZIBs) is hindered by interfacial processes that occur at the cathode. Because of capacity fading and poor rate capability, manganese dioxide (MnO_2_) cathodes are also negatively affected. Here, the novel application of in situ grazing incidence x‐ray absorption spectroscopy (GI‐XAS) to investigate the cathode‐electrolyte interfacial dynamics in a MnO_2_ cathode ZIB is demonstrated. By using a low‐incidence angle X‐ray beam to selectively probe the cathode surface, in situ changes are captured in the MnO_2_ oxidation state and local structure during charge–discharge. Results reveal that MnO_2_ undergoes a dissolution‐redeposition mechanism at the interface. During discharge, Mn^4+^ is reduced to Mn^3+^ and further to Mn^2+^ species that migrate into the electrolyte. Whilst charging, these Mn^2+^ species form a transient Mn^2+^‐rich layer on the cathode surface. This surface layer impedes Zn^2+^ transport and causes increased overpotential, correlating with capacity decay. Such interfacial transformations are fully reversible in the bulk of the cathode but only partially reversible at the surface, thus leading to residual Mn^2+^/ Mn^3+^ species after recharge. The findings provide direct evidence of cathode surface reconstruction as a potent contributor to performance degradation.

## Graphical abstract

This study demonstrates the first in situ application of grazing incidence X‐ray absorption spectroscopy (GI‐XAS) to probe interfacial degradation in MnO_2_ cathodes of zinc‐ion batteries. By varying the incidence angle, surface‐ and bulk‐specific redox changes are revealed, highlighting Mn dissolution and incomplete reoxidation as key factors driving capacity fading.

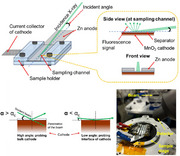

## 1 Introduction

Over the past decade, rechargeable ZIBs have garnered significant attention as a safe and cost‐effective energy storage technology for large‐scale applications.^[^ ^1^, ^2^ ^]^ The concept of a rechargeable ZIB using intercalation chemistry was first demonstrated in the early 2010s.^[^ ^3^ ^]^ Since then, the field has rapidly developed. A typical ZIB consists of a zinc metal anode, a Zn‐containing electrolyte, often aqueous or mildly acidic, and a metal oxide cathode capable of reversibly inserting Zn^2+^ ions.^[^ ^4^ ^]^ Of the various cathode materials, manganese dioxide (MnO_2_) is one of the most attractive due to its high theoretical capacity, abundance, and environmental benignity.^[^ ^1^, ^5^ ^]^ Yet, realizing an effective and practical Zn‐MnO_2_ battery remains challenging.^[^ ^6^, ^7^ ^]^ ZIBs still face bottlenecks in rate performance and cycling stability. Such constraints have impeded their commercialization so far. These issues have been linked to complex interfacial processes and phase changes during Zn^2+^ insertion and extraction.^[^ ^5^ ^]^

Of late, research has targeted multiple strategies to improve ZIB performance, including developing advanced cathode materials and formulating electrolytes that protect the zinc anode via artificial solid‐electrolyte interphases.^[^ ^8^, ^9^ ^]^ To mitigate problems like hydrogen evolution at the Zn anode, our group has focused on nonaqueous and hybrid electrolytes. We demonstrate that a deep eutectic solvent‐based electrolyte and other aprotic systems: choline chloride‐urea^[^ ^10^ ^]^ or dimethyl sulfoxide^[^ ^11^ ^]^ electrolytes, effectively suppress water‐induced side reactions, enabling stable Zn plating/stripping. Despite stabilizing the anode, we note that these purely nonaqueous electrolytes yielded only moderate capacities (≈90 mAh g^−1^) with MnO_2_ cathodes. Subsequently, we found that when a small amount of water (5 wt.%) is introduced into a DMSO‐based electrolyte, the capacity of MnO_2_ cathode dramatically improved, reaching ≈200 mAh g^−1^. The presence of water appears to facilitate Zn^2+^ insertion kinetics or activate additional redox processes in MnO_2_.^[^ ^12^ ^]^ In these “wet” nonaqueous electrolytes, we show that pre‐intercalating species like ammonium ions into δ‐MnO_2_ can create hydrogen‐bond networks that enhance capacity up to 240–250 mAh g^−1^.^[^ ^13^ ^]^ These improvements, however, come at the cost of long‐term stability. MnO_2_ cathodes in such electrolytes still exhibit gradual capacity degradation over extended cycles and poor rate performance (rapid capacity fade at high discharge rates). The root causes of these failure modes remain to be fully understood.

A growing body of evidence suggests that interfacial mechanisms at the cathode‐electrolyte boundary play a pivotal role in the degradation of MnO_2_ cathodes.^[^ ^5^, ^14^ ^]^ For instance, MnO_2_ in mildly aqueous electrolytes is known to undergo a dissolution‐precipitation reaction.^[^ ^15^, ^16^ ^]^ First, during discharge, Mn^4+^ is reduced to Mn^3+^. Then, a fraction of Mn^3+^ can disproportionate into Mn^4+^ (remaining in the solid) and soluble Mn^2+^ that dissolves into the electrolyte. Next, the dissolved Mn^2+^ may diffuse away from the cathode, causing active material loss, or later redeposit on the electrode as various Mn^2+^/ Mn^3+^ compounds upon charging.^[^ ^5^ ^]^ This mechanism has been directly visualized in aqueous ZIBs using in situ Raman spectroscopy. Wu et al. (2021)^[^ ^17^ ^]^ observed that, during discharge, MnO_2_ cathodes form soluble Mn^2+^ species, which subsequently redeposit as a surface layer on charge. Such processes can create an insulating passivation film, which can increase cell impedance and obstruct Zn^2+^ diffusion. In the literature, to disentangle these processes, alternative Zn^2+^ storage mechanisms like solid‐state intercalation or conversion reactions have been proposed.^[^ ^18^, ^19^ ^]^ Difficulties can arise, especially for δ‐MnO_2_ (birnessite), which is a layered MnO_2_ polymorph with low crystallinity.^[^ ^20^ ^]^ Birnessite‐type MnO_2_ often undergoes only subtle structural changes upon Zn^2+^ intercalation, making it hard to track phase transitions by X‐ray diffraction (XRD).^[^ ^9^ ^]^ Its inherently disordered, turbostratic structure yields broad XRD peaks and can accommodate Zn^2+^ (and H^+^) insertion with minimal long‐range order changes.^[^ ^21^, ^22^ ^]^ Conventional ex‐situ or bulk analytical techniques may not capture the dynamic interfacial phenomena in this cathode material.

Prior studies of MnO_2_ cathodes have relied on ex‐situ characterization or in situ diagnostics that probe the bulk of the material.^[^ ^23^ ^]^ While these approaches provide valuable information on average oxidation states and phase composition, they may miss surface‐localized reactions.^[^ ^24^ ^]^ For instance, in situ XRD can detect the formation of Zn‐containing phases, such as Zn birnessite or ZnMn_2_O_4_ spinel, in MnO_2_ cathodes. However, XRD lacks sensitivity to amorphous surface layers or dissolved species. In situ Raman spectroscopy can directly monitor certain molecular species and reaction intermediates on the cathode.^[^ ^17^ ^]^ Indeed, Raman spectroscopy has identified Mn^3+^ and Mn^2+^ species during ZIB operation. However, Raman spectroscopy is affected by issues like fluorescence background, spot‐to‐spot variability, and laser‐induced changes, leading to reproducibility challenges.^[^ ^25^ ^]^ One review has noted that in situ Raman results for electrocatalytic systems can be inconsistent and require careful interpretation.^[^ ^26^, ^27^ ^]^ More importantly, Raman spectroscopy and similar techniques cannot quantitatively determine the oxidation state of Mn or its local atomic environment with high precision.^[^ ^28^, ^29^ ^]^ For example, as shown in our Raman results (Figure S1, Supporting Information), similar issues are clearly observed in our study. This is a critical gap, as the Mn valence directly reflects the charge compensation mechanism during Zn^2+^ insertion, whether by Mn redox or other processes.

To address these challenges, we explore in situ grazing‐incidence X‐ray absorption spectroscopy (GI‐XAS) as a new method to investigate the cathode‐electrolyte interface in ZIBs. XAS, especially at the Mn K‐edge (≈6.5 keV), is a powerful tool for probing the oxidation state and local coordination structure of Mn in MnO_2_.^[^ ^30^ ^]^ In traditional transmission or fluorescence XAS measurement, the signal averages over the entire electrode thickness.^[^ ^31^ ^]^ By employing GI geometry and directing the X‐ray beam at a very shallow angle (OFN the order of 0.1°) relative to the electrode surface, one can tune the penetration depth of the X‐rays to predominantly probe the near‐surface region (a few to hundreds of nanometers).^[^ ^32^ ^]^ GI‐XAS thus offers depth‐resolved chemical information. At low incidence angles, the data is surface‐sensitive. At higher angles, the bulk of the electrode is probed.^[^ ^33^ ^]^ This depth adjustability is ideal for studying interfacial mechanisms in batteries, where surface and bulk may undergo different changes.

Notably, to the best of our knowledge, in situ GI‐XAS has not previously been reported for MnO_2_ cathodes or for any other battery cathodes.^[^ ^34^, ^35^ ^]^ Our study, therefore, provides an original demonstration of the potential of this approach in the context of zinc battery research. However, applying GI‐XAS in an electrochemical cell is non‐trivial. In situ GI setups must accommodate the small incident angles and often require custom cell designs. Techniques like grazing‐incidence X‐ray scattering or reflectometry have been highlighted as promising for battery interphase studies, but are challenging to implement in the in situ mode.^[^ ^36^ ^]^ Here, we overcome these challenges by designing a specialized in situ cell and leveraging a high‐flux synchrotron X‐ray beamline to perform GI‐XAS on a working Zn‐MnO_2_ battery.

In this paper, we present an in situ GI‐XAS investigation of a δ‐MnO_2_ cathode in a wet nonaqueous Zn‐ion battery (5% water in dimethylformamide with Zn^2+^ salt). We monitor the Mn K‐edge X‐ray absorption near‐edge structure (XANES) spectra at two extreme incidence angles, corresponding to the cathode surface and bulk, respectively, as the cell is charged and discharged. By comparing the surface‐sensitive and bulk‐sensitive spectra, we directly observe the development of a Mn^2+^‐rich surface layer during discharge and its partial reversion during charge. We correlate these spectral changes with the cell's electrochemical behavior to elucidate how the interfacial Mn chemistry influences the overall performance. The insights from this in situ GI‐XAS study shed light on the long‐term degradation mechanisms of MnO_2_ cathodes, identifying the formation of a Mn‐contained passivating layer as a culprit for increased polarization and incomplete capacity recovery. Our findings underscore the importance of understanding and controlling interfacial reactions in ZIBs. Moreover, this work establishes in situ GI‐XAS as a valuable methodology for battery research, opening up new avenues to study solid‐electrolyte interphases and other interfacial phenomena with element‐specific sensitivity and depth resolution. Such knowledge is crucial for designing strategies to prolong cycle life and enhance the rate capability of next‐generation zinc‐ion batteries.

## 2 Material and Experimental Setup

### 2.1 Materials

All chemicals were used as received without further purification. Potassium permanganate (KMnO_4_, 99.0%) was purchased from KemAus. Both Manganese (II) sulfate monohydrate (MnSO_4_·H_2_O, 99%) and N‐methyl‐2‐pyrrolidone (NMP, 99.5%) were obtained from Qrec. N,N‐Dimethylformamide (DMF, 99.7%) was purchased from Supelco. Zinc trifluoromethanesulfonate (Zn(CF_3_SO_3_)_2_, 98%, abbreviated Zn triflate) was bought from Sigma‐Aldrich. Poly(vinylidene difluoride) (PVDF, Solef 5130) binder was provided by Solvay. Ketjenblack EC‐600JD carbon (conductive additive) was obtained from Cabot Corp. Graphite foil (Grafoil, 25 µm thickness) current collector was sourced from Shenzhen 3KS Electronic Materials. A microporous hydrophilic polypropylene separator (40 µm thickness, for Zn‐Ni batteries) was obtained from Shandong Gold Yunji. A 5 µm thin polypropylene film (Cole‐Parmer 3520‐SSP) was used as an X‐ray transparent window. High‐purity zinc foil (99.99%, 50 µm thick) for the anode was purchased from Shandong AME Energy.

### 2.2 MnO_2_, Synthesis and Material Characterizations

The cathode material studied is a potassium birnessite form of MnO_2_ (layered δ‐MnO_2_). We synthesized K‐birnessite via a facile hydrothermal reduction of KMnO_4_ by Mn^2+^, adapted from the literature.^[^ ^10^, ^37^ ^]^ In a typical synthesis, 1.896 g of KMnO_4_ was dissolved in 70 mL of deionized water under vigorous stirring. Then, 0.334 g of MnSO_4_·H_2_O was added to this purple KMnO_4_ solution, initiating a redox reaction that produced MnO_2_. The mixture was stirred for 30 min to ensure complete reaction, yielding a brown suspension. The suspension was transferred to a Teflon‐lined autoclave and heated at 160 °C for 17 h. After naturally cooling, the solid precipitate was collected by filtration, washed with water, and dried under vacuum at 60 °C overnight. The resulting MnO_2_ powder (denoted K‐MnO_2_) was characterized to confirm its structure and morphology by using X‐ray diffraction (XRD; Aeris Benchtop, Malvern PANalytical; Cu‐Kα; 40 kV, 15 mA), field‐emission scanning electron microscopy (FE‐SEM; QuantaTM 250 FEG (FEI)), energy‐dispersive X‐ray spectroscopy (EDS; PentaFET, Oxford Instruments), and transmission electron microscopy (TEM; JEOL Model JEM‐2100).

### 2.3 Preparation of the MnO_2_ Cathode and Wet Nonaqueous Electrolyte

For the electrochemical tests, MnO_2_ cathodes were prepared in the form of a composite coating on graphite foil. The MnO_2_ powder was mixed with conductive carbon (Ketjenblack) and PVDF binder in various weight ratios, using NMP as the solvent to make a slurry. We explored different MnO_2_:carbon:binder ratios of 70:20:10, 45:45:10, and 15:75:10 (by weight) to study the effect of MnO_2_ loading on XAS measurements. Unless otherwise specified, the cathode used for in situ experiments had the standard high active material content (70% MnO_2_) to represent a realistic battery electrode. The mixture was thoroughly dispersed by alternating magnetic stirring and ultrasonication until a homogeneous ink was obtained. The slurry was then doctor‐bladed onto a 25 µm graphite foil current collector to form a uniform film. To evaporate NMP, the coated sheet was dried at 80 °C for 3 h. Then, the sheet was gently calendered (rolled) to improve particle contact and electrical conductivity. A final drying at 60 °C, under vacuum overnight, removed residual solvent. The resulting electrode had a MnO_2_ loading of ≈2 mg cm^−2^, a coating thickness of ≈10 µm, and a compressed density of ≈2.86 g cm^−3^. This relatively thin electrode is beneficial for X‐ray penetration and minimizes self‐absorption, albeit at the expense of total capacity per area.

Prior to cell assembly, the cathodes were kept in a desiccator to avoid moisture uptake. The electrolyte was a “wet” nonaqueous Zn^2^⁺ solution consisting of Zn triflate salt in a DMF‐water mixed solvent. We prepared it by first making a stock solution of 5 wt.% deionized water in DMF. Zinc triflate was then dissolved in this solvent to a concentration of 0.5 m. The mixture was stirred at room temperature (≈25 °C) for 15 min until the salt fully dissolved, yielding a clear. The small addition of water greatly enhanced Zn^2+^ transport and the electrochemical activity of the MnO_2_ cathode, as demonstrated in our previous work.^[^ ^12^ ^]^ The predominantly aprotic DMF medium is seen to suppress the parasitic hydrogen evolution at the Zn anode.^[^ ^38^ ^]^ To ensure full wetting, the separator (hydrophilic PP, 40 µm) was soaked in this electrolyte overnight before cell assembly.

### 2.4 In Situ GI‐XAS Cell Configuration

**Figure 1:**
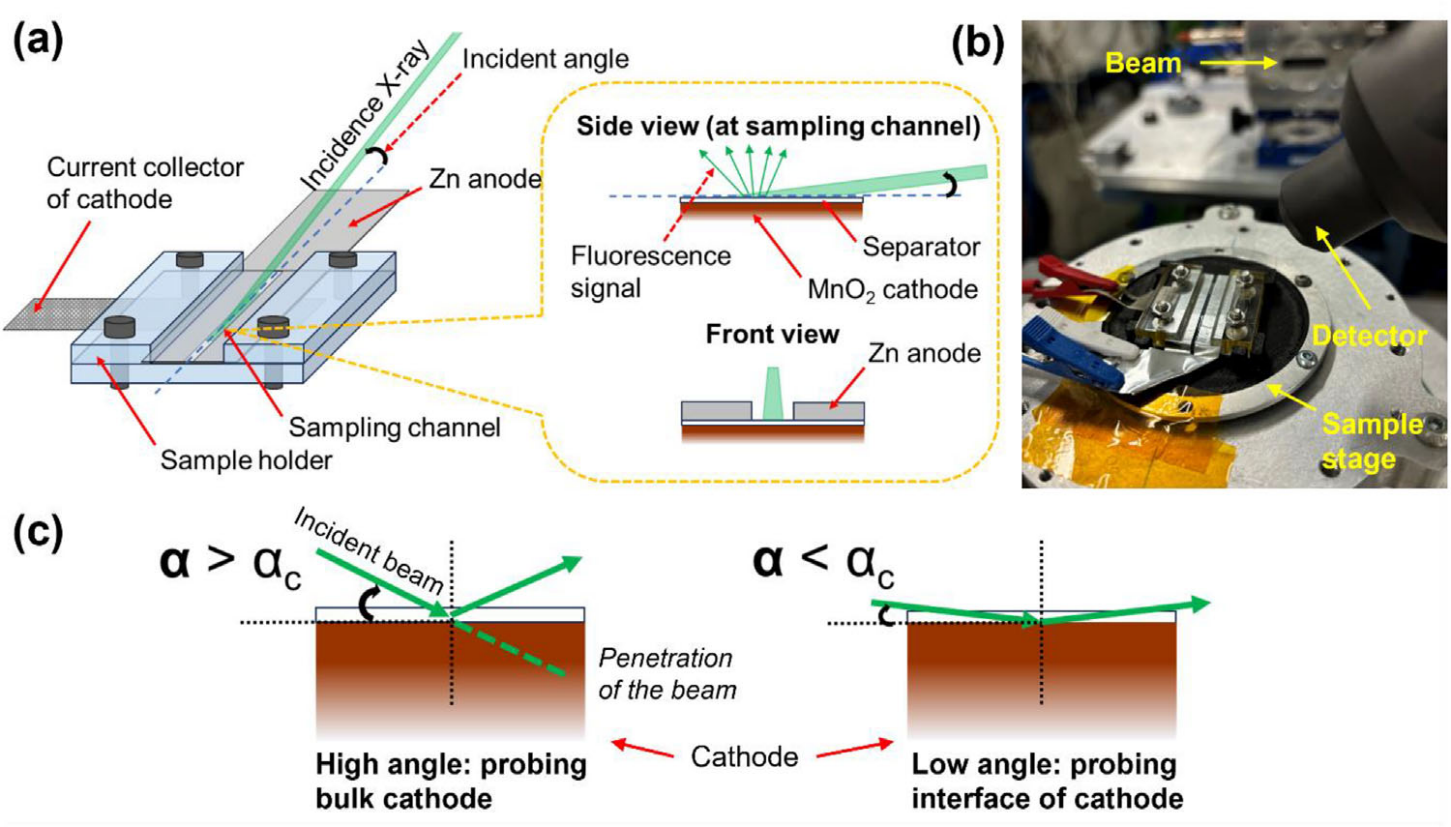
a) Schematic illustration of the cell for GI‐XAS, b) The cell mounted at the diffractometer, and c) Schematic illustration of X‐ray beam penetration into the sample at different incidence angles.

For the in situ GI‐XAS measurements, a custom two‐electrode cell was designed to resemble a “sandwich” structure while allowing the X‐ray beam to impinge at a shallow angle on the cathode. In **Figure** **1**a, the cell architecture is displayed. The MnO_2_ cathode (coated on graphite foil) is placed at the bottom, acting as the working electrode. The zinc foil anode (counter and reference electrode) is on the top side. The separator soaked in electrolyte is sandwiched between the two electrodes. The cell components are held together by a plastic sample holder with stainless‐steel bolts, providing a leak‐tight yet X‐ray accessible setup. A key feature of the design is that the Zn foil anode has a narrow slit (gap) of ≈1.5 mm width (and >3 cm length) situated in the region above the cathode. This slit serves as a window for the X‐ray beam: the low‐angle X‐ray can pass through the gap and strike the cathode surface without attenuation by the zinc anode. The slit is sufficiently long to cover the X‐ray beam footprint during incidence and allows fluorescence detection from the cathode beneath. In Figure 1b, a photograph of the assembled cell mounted on the diffractometer stage at the beamline is given.

In situ GI‐XAS experiments were carried out at the SIRIUS beamline of Synchrotron SOLEIL (France). SIRIUS beamline is specifically designed for in situ X‐ray diffraction and spectroscopy studies of materials, which makes it well‐suited for our in situ battery cell.^[^ ^39^ ^]^ The cell was mounted on a 6‐axis sample tower of a 7‐circle Newport diffractometer. The incident X‐ray beam, of energy tunable around the Mn K‐edge (6540 eV), was focused on the vertical plane and delimited by slits in a horizontal to obtain a spot size of ≈0.5 × 0.7 mm^2^ at the sample level. We used a beam flux of ≈1.3 × 10^11^ photons/s, high enough to yield strong fluorescence signals even at low angles. Alignment of the cell was achieved by scanning the incidence angle while monitoring the Mn Kα fluorescence signal, ensuring the beam struck the cathode at the center of the slit window. The fluorescence from the sample was collected by a four‐element silicon drift detector (SDD) positioned at 90° to the beam and at 45° above the sample plane, which captured the Mn k‐alpha X‐ray fluorescence from the sample A Pilatus 1 m detector mounted on the diffractometer detector arm was also used for assisting with the cell alignment. The incidence angle (α) of the beam relative to the cathode plane could be precisely varied using the diffractometer actuators, allowing us to probe different depths of the cathode.^[^ ^40^ ^]^

During in situ measurements, the Zn‐MnO_2_ cell was connected to a potentiostat (BioLogic SP‐300) in a two‐electrode configuration (MnO_2_ working, Zn counter/reference). To change the state of the battery, galvanostatic charging/discharging at a moderate current rate (≈1C) was employed, meaning the cell was charged/discharged at a constant current while we intermittently paused to record XAS spectra. First, the cell was charged to 100% state of charge (SOC) to obtain a fully charged baseline (point 1). Then, we discharged in steps to 75%, 50%, 25%, and 0% SOC, pausing at each point (points 2–5) to hold the cell and collect XAS spectra. Subsequently, the cell was charged back up in steps: 25%, 50%, 75%, 100% SOC (at points 6–9, respectively) with XAS measured at each. Holding the cell at each point for spectrum acquisition, taking ≈5–10 min per spectrum, is seen to introduce brief relaxations in the voltage. This method ensured we captured XAS at defined states of charge.

Throughout these steps, the cell voltage was recorded as a function of time to correlate with the XAS data (Figure 4a). All XAS spectra were collected in fluorescence mode due to the necessity to probe the cation surface and not interfere with the electrical cycles (transmission and total electron yield modes not possible). As detailed below in Section 3.2, two incidence angles were used for each point: a low angle to probe the cathode surface/interface, and a higher angle to probe the bulk of the cathode, as illustrated in Figure 1c. These angles were chosen based on prior alignment and calculation of X‐ray penetration depth for probing either the interfacial region or the full thickness of the electrode, averaging the bulk MnO_2_ response. By comparing the XANES spectra from these two angles at various states of charge, we were able to discern differences between surface and bulk redox processes of Mn. XANES results were processed using Athena (Demeter version 0.9.26).^[^ ^41^ ^]^

## 3 Results and Discussion

### 3.1 Powder Characterization of K‐MnO_2_

**Figure 2:**
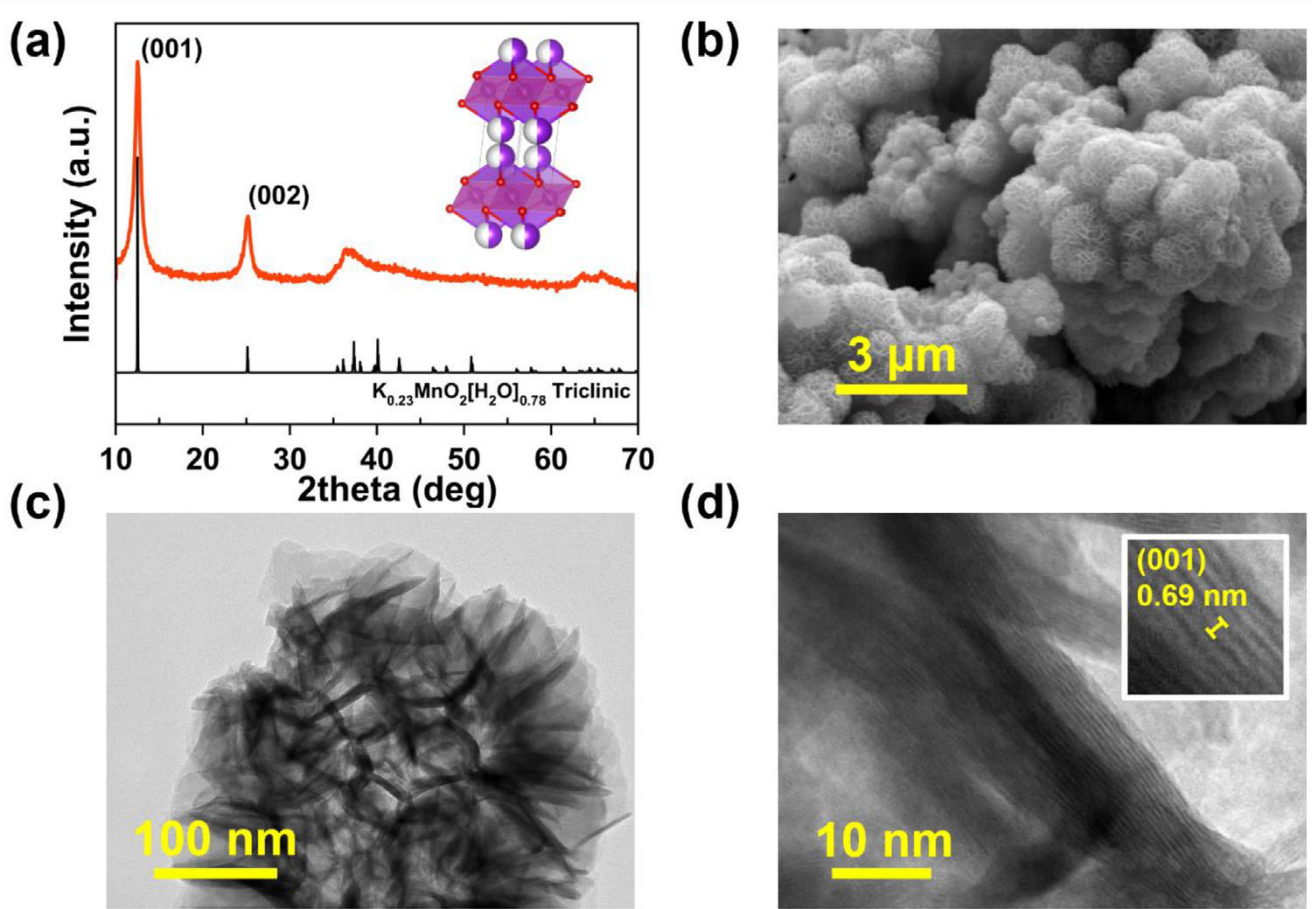
Powder characterization results of K‐MnO_2_: a) XRD spectrum and reference structure: K_0.23_MnO_2_·0.76H_2_O,^[^ ^42^ ^]^ b) FE‐SEM image, TEM images at: c) 100,000x, and d) 800,000x.

In **Figure** **2**, the physical characterization of the K‐MnO_2_ powder is presented. In Figure 2a, the XRD pattern reveals two distinct diffraction peaks at 2θ ≈ 12.3° and 24.9°, along with broad peaks in the ranges of 35–38° and 62–68°. These peaks match well with standard birnessite δ‐MnO_2_ (K_0.23_MnO_2_·0.76H_2_O).^[^ ^42^ ^]^ The broad nature of the peaks indicates the material is nanocrystalline with turbostratic disorder, typical of birnessite.^[^ ^43^ ^]^ This behavior poses a challenge for crystallographic analysis using XRD, particularly due to the overlapping peaks in the 35–38° region, which correspond to intralayer reflections of the MnO₆ octahedral framework and remain poorly resolved. The interlayer spacing calculated from the (001) peak is ≈7 Å, consistent with birnessite containing interlayer water/K⁺ ions.^[^ ^44^ ^]^ The morphology of MnO_2_ was examined via electron microscopy. In Figure 2b, FE‐SEM exhibits a nanoflower‐like architecture, in which the particles (5–10 µm agglomerates) are composed of petal‐shaped nanosheets. In Figure S2 and Table S1 (Supporting Information), EDS analysis confirms the presence of K, Mn, and O in the sample, with an approximate composition of K_0.3_MnO_1.99_ (atomic ratio). The composition is close to the expected birnessite formula reported in the literature.^[^ ^43^, ^44^ ^]^

In Figure 2c,d, TEM images further show that each “petal” is a stack or bundle of thin MnO_2_ layers (nanosheets) on the order of a few nanometers thick. The high‐magnification TEM (Figure 2d) shows a layered structure with an interlayer gap of ≈7 Å, in agreement with the XRD result. Such a hierarchical nanoflower morphology with ultrathin layers is known to be beneficial for ion intercalation, as it provides a high surface area and short solid‐state diffusion paths.^[^ ^45^ ^]^ Similar nanoflower δ‐MnO_2_ structures have been reported to enhance Zn^2+^ storage by offering abundant insertion channels and defect sites.^[^ ^46^ ^]^ Characterization confirms that we have successfully synthesized K‐birnessite MnO_2_ with the desired structure and microstructure for use as a ZIB cathode material.

### 3.2 Transmission, Penetration Depth, and Grazing Incidence Angle

In GI‐XAS experiments, it is important to understand how far X‐rays can penetrate into the sample at different incident angles, and to account for any attenuation by cell components, such as the thin window and separator. Here, the cathode is covered by a polypropylene separator (40 µm) plus the 5 µm PP film window. These thicknesses are relevant, and these elements absorb an important percentage of the incident beam. However, the critical angle for total reflection of polypropylene is smaller than the theoretical one for MnO_2_‐based cation, due to the lower density.^[^ ^47^ ^]^ Therefore, choosing an incidence angle larger than the critical angle of polypropylene, the X‐rays can nonetheless penetrate enough in the separator and reach the cathode, allowing its characterization, thanks also to the high flux available at the beamline. This means that our in situ cell design is X‐ray transparent enough to yield strong fluorescence signals from the MnO_2_, given the high flux available. Then, we calculated the penetration depth of the X‐rays into the MnO_2_ cathode as a function of incidence angle (α).

**Figure 3:**
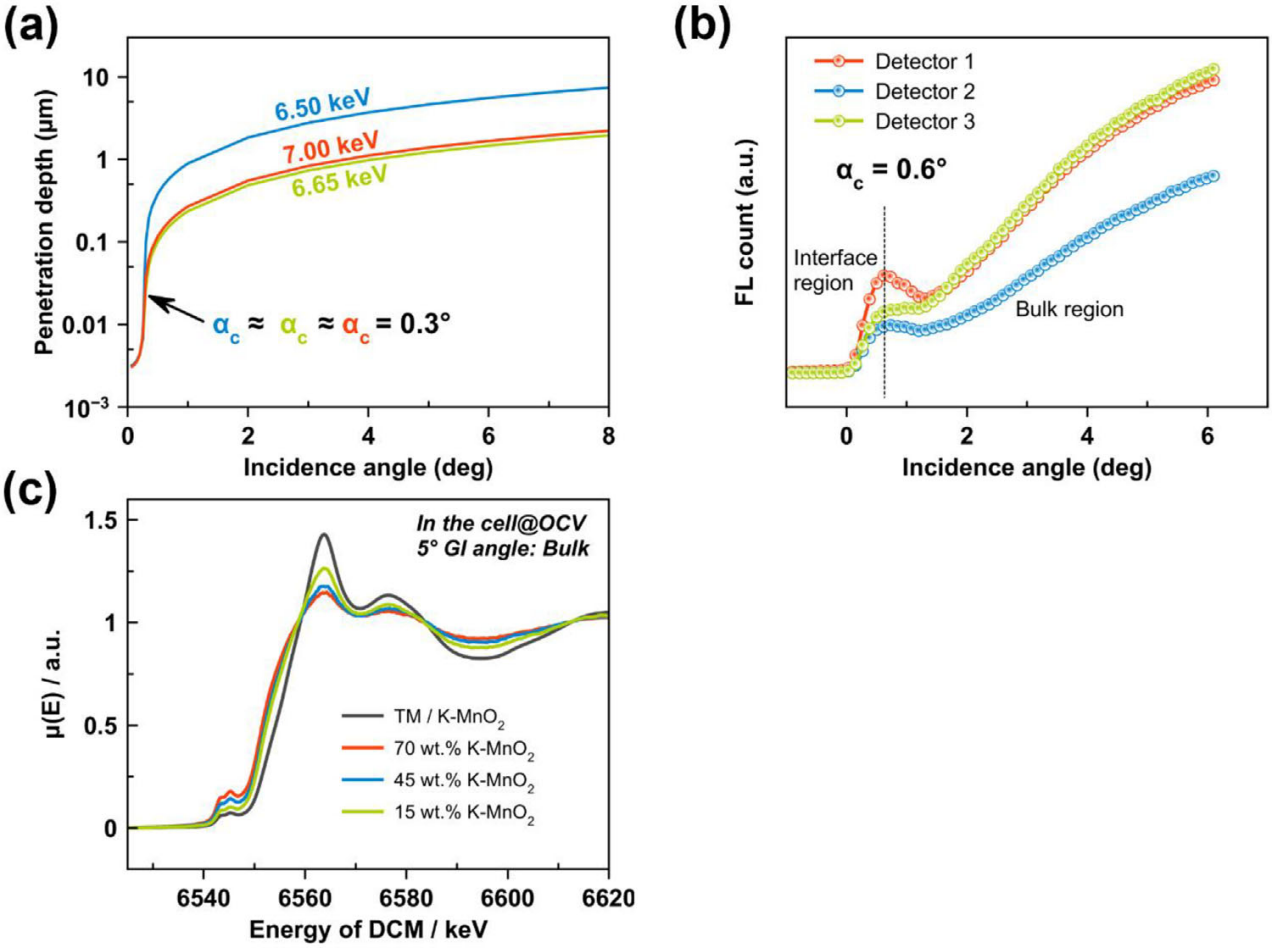
a) Theoretical penetration depth of the X‐ray beam into the cathode, b) Count of the fluorescent Mn signal from the sample versus incidence angle of the X‐ray beam at an energy of 7 keV, and c) XANES spectra of MnO_2_ cathode at different fractions of MnO_2_‐conductive carbon‐binder: 70‐20‐10, 45‐45‐10, and 15‐75‐10 by weight.

X‐ray penetration depth is defined as the distance at which X‐ray intensity falls to 1/e of its surface value. Thus, it depends on the incident angle, X‐ray energy, and the complex refractive index (density and composition) of the sample. Employing the known density of our electrode (2.86 g cm^−3^) and the chemical formula of birnessite, we employed the calculation tool in the Center for X‐Ray Optics (CRXO) database to approximate penetration depth versus angle at relevant energies.^[^ ^48^ ^]^ In **Figure** **3**a, the results are shown. At very shallow angles, below the critical angle for total external reflection, X‐rays can only penetrate a few tens of nanometers, effectively sampling the surface. We identified a critical angle α_c_ ≈ 0.3° at ≈6.5 keV for our MnO_2_, beyond which X‐ray penetration increases rapidly: X‐rays begin entering the bulk. In the angle range of 0°–0.6°, penetration depth is seen to rise sharply from a few nm up to a few micrometers. For instance, at α = 0.2° (below α_c_), penetration is on the order of ≈10–20 nm. At α = 0.4° (just above α_c_), it reaches ≈0.1 µm; and at α = 1°, it is a few microns. At energies just above the Mn K‐edge, e.g., 6.6‐7.0 keV, it is noted that penetration is slightly reduced because Mn absorption increases.

To compare these calculations with the experiment, we conducted angle‐dependent X‐ray fluorescence scans on the assembled cell prior to cycling. When the cell was at open‐circuit at different charge/discharge states, Mn Kα fluorescence intensity was recorded as a function of the incidence angle. Due to the geometry of the cell and the non‐flat character of the cathode and covering layers, it is not possible to identify a clear reflected x‐ray beam and use reflectivity curves for the determination of the critical angle, as classically done.^[^ ^49^ ^]^ However, we can still look for the incipient Mn fluorescence yield, increasing the incidence angle from zero angle, to identify the value at which the x‐ray beam starts penetrating the Mn‐containing cathode. In Figure 3b, the Mn fluorescence counts exhibit a characteristic curve. At very low angles, the signal is low. Then, it rises steeply, reaching a peak ≈0.6°. Afterward, it gradually increases or levels off. A similar trend for fluorescence has been observed in total x‐ray reflection studies of atoms embedded in a near‐surface layer, such as in thin homogeneous contaminant layers at or slightly below the surface in semiconductors.^[^ ^50^, ^51^ ^]^ In these examples, the enhancement of x‐ray fluorescence is due to the excitation of a stationary wave field in total reflection conditions, and the observed peak indicates the position of the critical angle.^[^ ^52^, ^53^ ^]^ Even if in our case we are not in the presence of a flat substrate like in the examples given above, we believe that the angle‐dependent trend of the X‐ray fluorescence at grazing angles can be explained by a similar mechanism and that the peak observed ≈0.6 deg in Figure 3b is related to the position of the critical angle for the MnO_2_/electrolyte system. Experimentally, the fluorescence signal shows a “semi‐circular” rise until ≈1.2° and then transitions to a linear regime beyond that. In Figure 3b, we mark the boundary between these regimes with a dashed line, distinguishing the surface‐sensitive zone (left of the line) from the bulk‐sensitive zone (right of the line). The critical angle observed is ≈0.6°, which is higher than the 0.3° predicted for an ideal dry MnO_2_ sample. This discrepancy is likely due to the presence of the electrolyte within the porous cathode and at the surface, which can alter the effective refractive index and X‐ray reflectivity. The electrolyte, mostly DMF/H_2_O, with low‐Z elements, would slightly increase the critical angle compared to a vacuum; other factors like surface roughness could broaden the transition. Moreover, due to the impossibility of using x‐reflectivity to measure the offset between the sample (theta) and detector (2 theta) angle of the diffractometer, the zero position of the angle in Figure 3b could be imprecise. Nonetheless, looking at the results presented in the following, the qualitative agreement with expectations seems good: angles below ≈0.5–0.6° predominantly probe the interface while angles above ≈1–2° penetrate the bulk. Our chosen angles for in situ measurements were α ∼0.4°, for interface and ≈6° for bulk, referring to the scale of Figure 3b. The angle of 0.4°, is lower than the critical one, and the X‐ray beam probes roughly the top 3–4 nm of the electrode (the interfacial region), whereas at 6°, the beam penetrates ≈10 µm in thickness. This procedure effectively averages the entire cathode, behaving like a transmission measurement through the full coating.

Thus, our choice of 0.4° as a “grazing angle” ensured we focused on the interfacial region, and6° provided true bulk information. With this understanding, we proceed to analyze the XAS spectra at these two angles during battery operation.

### 3.3 Effects of MnO_2_ Loading and Self‐Absorption on XAS Spectra

One challenge encountered in fluorescence‐mode GI‐XAS is the self‐absorption effect, especially for a concentrated element like Mn in a thick sample. Self‐absorption (also known as saturation) can distort the XANES (X‐ray absorption near‐edge structure) spectral shape when the fluorescent X‐rays are reabsorbed by the sample before escaping.^[^ ^54^ ^]^ This effect is more pronounced for high loading of the absorbing element and for detection angles that are not in normal‐incidence/ grazing‐exit geometry.^[^ ^55^ ^]^ In our case, the Mn content in the cathode is relatively high (70 wt.% in the standard electrode), and we work at grazing incidence, so some amplitude attenuation of the Mn K‐edge XANES is expected. We investigated this by measuring fluorescence XANES on cathodes (in the cell) with different MnO_2_ concentrations: namely, the 70%, 45%, and 15% MnO_2_ electrodes mentioned earlier.

In Figure 3c, the Mn K‐edge XANES of these samples in fluorescence yield (FY) mode are presented, compared to a “true” XANES measured in transmission (TM) mode on a dilute reference sample. It is significant that all the FY‐XANES spectra show a damping of features relative to the transmission spectrum. Distortion is worst for the highest MnO_2_ content (70%), where the white‐line intensity is suppressed, and the post‐edge oscillations are flattened. Reducing the MnO_2_ content to 45% and 15% incrementally improves the spectral fidelity, although even at 15% some deviation remains. This trend confirms that self‐absorption is substantial in our thick electrode case. In principle, one can apply mathematical corrections for self‐absorption or use an optimized geometry to minimize it.^[^ ^55^, ^56^ ^]^ In our in situ setup, however, we are constrained by the cell geometry and the need for a representative electrode. We decided to use the standard 70:20:10 electrode for most experiments to mimic real battery conditions, accepting some XANES distortion. The key point is that despite these distortions, the primary indicators of Mn oxidation state remain interpretable. For instance, the energy position of the XANES absorption edge (the threshold) still correlates with the Mn valence, and we can track shifts in the edge to see if Mn is being reduced or oxidized. The distortion is, in fact, expected to affect only the overall amplitude of the XANES features and not their position.

### 3.4 In Situ GI‐XANES: Surface Versus Bulk Interfacial Dynamics

**Figure 4:**
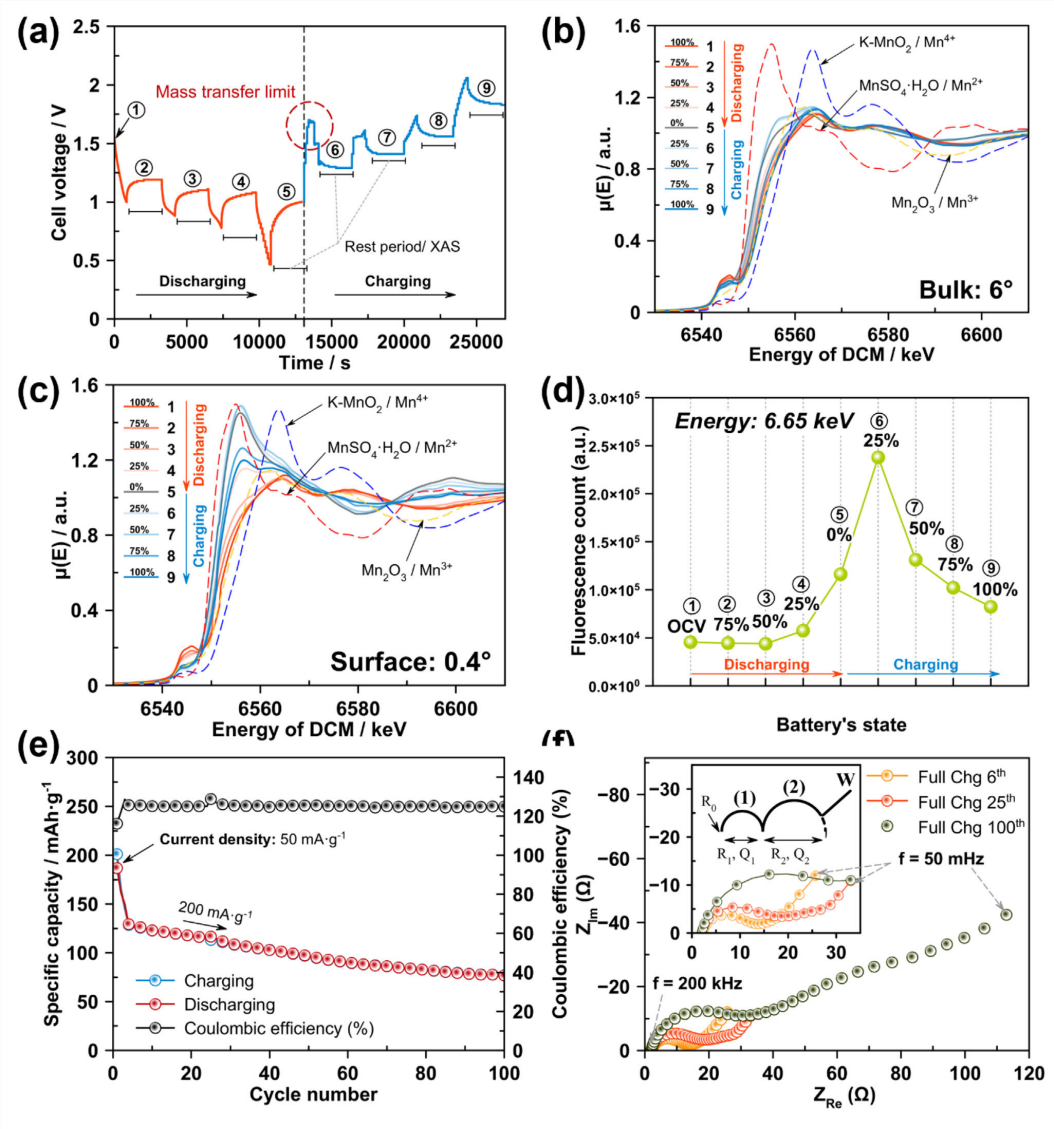
Recoded in situ data during 1 cycle of charging/discharging: a) Cell voltage versus time, b) Normalized XANES spectra of the bulk cathode (incidence angle: 6°), c) Normalized XANES spectra of the interface of the cathode (incidence angle: 0.4°), d) Count of the Mn Kalpha fluorescence signal from the cell at different state of the battery, e) cycling test of the K‐MnO_2_ cathode in coin cell, and f) EIS spectra of the coin cell after: 6, 25 and 100 cycles.

To monitor how the Mn oxidation state changes in the bulk of the cathode versus the surface/interface, during one charge–discharge cycle, we performed in situ GI‐XAS on the Zn‐MnO_2_ cell. The cell was first charged to 1.9 V (100% SOC) and then discharged to 1.0 V (0% SOC), and finally recharged to 1.9 V. In **Figure** **4**a, the voltage profile is shown. A timeline for XAS data points is illustrated. The Zn‐MnO_2_ cell exhibits a typical discharge plateau ≈1.3–1.1 V and a charge plateau ≈1.7–1.9 V, consistent with Zn^2^⁺ insertion into and extraction from MnO_2_. The numbered markers on the curve correspond to the states at which XANES spectra were collected: points 1 through 9 as described earlier. In Figure 4b, the Mn K‐edge XANES spectra collected at a high incidence angle (≈ 6°) are displayed: the bulk cathode response is represented. At the initial state of the battery (point 1), the Mn K‐edge position was ≈6540 eV, indicating an average Mn oxidation state near +4. As the cell discharged, points 2, 3, 4, and 5 are seen to correspond to 75%, 50%, 25%, and 0% SOC. The XANES edge gradually shifts to lower energy.

At the fully discharged state (point 5), the edge shifted by a few eV, and the white‐line intensity, the peak just after the edge, is slightly diminished. These changes signify that during discharge, Mn is being reduced from Mn^4+^ toward Mn^3+^. Indeed, the spectrum at point 5 closely resembles that of a Mn^3+^ reference, i.e., Mn_2_O_3_. Although the spectrum does not shift all the way to a pure Mn^3+^ edge, this outcome is expected since not all Mn^4^⁺ reduce to Mn^3^⁺. In the bulk of the material, some fraction of Mn likely remains at a higher valence. Thus, the discharge product is a mixed‐valence compound. Nevertheless, the bulk trend is clear: discharging reduces Mn, and charging oxidizes Mn. On charging from point 5 back to 100% SOC (points 6–9), the edge moves back to higher energy. At the end of the charge, point 9 overlaps point 1 (Figure 4b). In essence, XANES spectrum has essentially returned to its initial state, indicating that the bulk of MnO_2_ has been reoxidized to Mn^4+^. This reversible behavior in the bulk is consistent with our previous observations (Figure S3, Supporting Information), implying that the insertion of Zn^2^⁺ into MnO_2_ is a largely reversible redox process for the Mn centers, with Mn cycling between +4 and +3 oxidation states.^[^ ^12^, ^13^ ^]^

The surface‐sensitive XANES (incidence angle ≈ 0.4°) yielded strikingly different insights. In Figure 4c, the Mn K‐edge XANES spectra, at the same series of states (points 1–9), are shown. Here, only the interface region of the cathode is examined. At the beginning (point 1), the surface XANES spectra are similar to the bulk, suggesting that after assembly and charging, the surface Mn is mostly in the Mn^4+^ state. During the early stages of discharge (points 2 to 3, 75% to 50% SOC), the surface XANES spectra change in parallel with the bulk. Hence, the edge shifts slightly lower, implying Mn reduction to ∼Mn^3+^. Up to 25% SOC (point 4), there are no obvious divergences between surface and bulk spectra: both indicate gradual reduction. However, at the fully discharged state (point 5, 0% SOC), the surface XANES spectra show a dramatic change. The white‐line peak intensity increases sharply, and the edge position shifts significantly lower. In fact, the spectrum at point 5, on the surface, has a markedly different shape. It resembles the signature of the Mn^2+^ species. The pre‐edge feature and edge energy at point 5 align with what is expected for Mn^2^⁺. For example, Mn^2+^ in aqueous solution, MnO, or Mn(OH)_2_ show such spectra.^[^ ^57^, ^58^ ^]^ This observation indicates that, at the cathode interface, a substantial portion of Mn has been reduced all the way to the +2‐oxidation state at full discharge. In contrast, Mn in the bulk is mostly limited to +3. The emergence of Mn^2+^ at the surface implies dissolution or a process of phase change viz. Mn^2+^ compounds. Likewise, it is seen that Mn^2+^ ions or sparingly soluble Mn were not part of the original solid MnO_2_ phase.

To corroborate this, we measured the intensity of Mn fluorescence at the surface as a function of state of charge (Figure 4d). As a result, the Mn signal at the interface remains roughly steady at 5 × 10^4^ counts throughout points 1–4. Then, it jumps almost 2.6‐fold at point 5. Consequently, it reaches ≈2.4 × 10^5^ counts at point 6, which is far beyond the initial level. This huge increase in Mn fluorescence at the surface likely means that Mn has migrated out of the dense oxide lattice into a form that yields stronger fluorescence. One interpretation is that Mn^2^⁺ ions have leached into the electrolyte near the interface. Dissolved Mn^2^⁺ in the liquid just above the cathode can produce a strong fluorescence signal since they are more surficial and, therefore, at the same time better probed by the grazing incidence beam and less affected by self‐absorption. The presence of dissolved Mn^2+^ in the electrolyte was further confirmed by ICP‐OES analysis: details of the test are provided in the Figure S4 (Supporting Information).

Another possibility is the formation of a concentrated Mn‐containing layer on the surface that is effectively “seen” as a higher Mn concentration by the X‐ray beam. In either case, the surge in signal strongly suggests Mn dissolution from the cathode at deep discharge. This outcome aligns with the known dissolution‐redeposition mechanism in aqueous Zn‐MnO_2_ systems.^[^ ^59^ ^]^ In our cell, even though the electrolyte is mostly organic, the addition of 5% water can enable similar chemistry. The reduction of MnO_2_ to Mn^2^⁺ likely involves water in the proton‐assisted MnO_2_ reduction reaction, such that MnO_2_ + 4H⁺ + 2e^−^ → Mn^2^⁺ + 2H_2_O.^[^ ^19^ ^]^ Zn^2^⁺ insertion may also produce local acidity or OH^−^ that facilitates Mn dissolution​. During the charging process, what happens after this point?

As we begin charging from the fully discharged state, cell voltage initially rises sharply around points 5 to 6 (Figure 4a). This high overpotential is a clue that something impedes the reaction. We propose that during the early charge, a Mn‐containing precipitate forms on the cathode surface. Specifically, the dissolved Mn^2^⁺ in the electrolyte can combine with any available OH^−^, from side reactions or from the reduction process, forming an insoluble Mn layer on the cathode. Alternatively, Mn^2^⁺ may directly redeposit as a Mn_3_O_4_ or Zn_x_MnO_y_·nH_2_O phase. The presence of Mn^2^⁺ and Mn^3^⁺ together with OH^−^ often leads to layered double hydroxides (LDH) or mixed‐valence hydroxides.^[^ ^60^ ^]^ For example, it is acknowledged that a mixture of Mn^2^⁺, Mn^3^⁺, and OH^−^ can form birnessite‐like or chalcophanite‐like phases (ZnMn_3_O_7_·3H_2_O) under some conditions.^[^ ^61^ ^]^ In our experiment, XANES spectrum at point 6, after a small amount of charge, still looks like Mn^2^⁺ with maybe a slight shift upward, indicating a Mn^2+^‐dominated surface.

At point 6, fluorescence intensity remains very high, almost as high as point 5 (Figure 4d). So, we infer that a Mn‐rich surface layer forms at the beginning of the charge, between points 5 and 6. Such an outcome coincides with the region of elevated voltage, which is consistent with a passivation layer that hinders electron transfer and Zn^2^⁺ de‐intercalation, causing polarization. As charging continues up to points 7 to 8, reaching ≈50–75% SOC, cell voltage behavior is seen to normalize. In Figure 4a, the curve resumes a typical slope after point 6. By point 8 (≈75% charged), the surface XANES spectrum has shifted to higher energy compared to points 5 and 6. It no longer matches a pure Mn^2^⁺ but appears to be of a mixed Mn^2+^/Mn^3+^ character. The edge is intermediate, and the white‐line intensity has decreased from its peak. Simultaneously, the Mn fluorescence count has dropped from its peak, at point 5/6, down to point 8, indicating that the initially formed Mn^2^⁺‐rich layer is being oxidized and possibly partially dissolving. This decrease in fluorescence could mean some Mn goes back into the bulk solid as Mn^3^⁺/Mn^4^⁺ or that the surface layer restructures into a less “thick” form. By the end of the charge at point 9 (100% SOC), the surface is not fully restored to the original Mn^4+^ state. XANES spectrum at the interface still shows a significant fraction of reduced Mn. The edge is found to be lower than point 1. This suggests the presence of Mn^3^⁺. Such an outcome indicates that reoxidation is incomplete, and some Mn^2^⁺/Mn^3^⁺ may remain in the surface layer even after the battery is charged.

To summarize, we have, in effect, a residual altered surface compound that did not convert back to pristine MnO_2_. Such a finding is crucial because it provides a direct microscopic explanation for capacity loss. If some Mn is permanently in a lower valent state at the surface, this implies that not all Zn^2^⁺ was removed from the cathode: some capacity is lost, forming a “dead” layer. In situ GI‐XAS reveals a clear sequence of interfacial events. 1) During discharge, Mn^4+^ in MnO_2_ is reduced to Mn^3+^, and at the surface, a further portion reduces to Mn^2+^, leaving the MnO_2_ lattice likely to dissolve into the electrolyte. 2) At full discharge, the cathode surface is enriched in Mn^2+^, either as adsorbed ions, or a newly formed Mn layer; the active material loses some Mn to the electrolyte. 3) Upon charging, the dissolved Mn^2+^ redeposits onto the cathode as a Mn^2+^‐containing layer, causing high polarization. 4) With continued charge, this layer partially oxidizes to higher‐valence Mn, and some Mn reintegrates, but reconstitution is not complete; a fraction of Mn remains in an altered state, and the original MnO_2_ framework at the surface is not fully restored. Notably, such interfacial phenomena are invisible in the bulk XAS or voltage alone. The bulk appears to be fully reversible. The voltage profile only shows a slight hump. Without GI‐XAS, one might assume that the cathode reaction is entirely a reversible Mn^4^⁺/Mn^3^⁺ phase transition, whereas in reality, side reactions, such as dissolution and passivation, are occurring. These insights underscore the significance of surface‐sensitive in situ techniques.

In Figure 4e, the cycling stability of the coin cell with the K‐MnO_2_ cathode is shown. After the current density is increased to 200 mA g^−1^ (4th cycle), the specific capacity of the cathode gradually declines, resulting in a capacity retention of ≈60%. As illustrated in Figure 4f and Table S2 (Supporting Information), electrochemical impedance spectroscopy (EIS) results reveal a significant increase in R_2_, representing the charge‐transfer resistance of the cathode, which reflects progressive degradation upon cycling: details of coin cell testing and EIS are given in SI. These findings suggest that interfacial irreversibility contributes to the accumulation of inactive phases during repeated cycling, leading to capacity fading and increased impedance over time.

In Figure S6a (Supporting Information), the XRD pattern of the cathode after 100 cycles shows additional features in the regions of 17–19°, 31–35°, and 57–62°, which is likely to be the Mn_3_O_4_ (COD‐9001302).^[^ ^62^ ^]^ However, the presence of Mn_3_O_4_ cannot be definitively confirmed due to its weak signal. As shown in Figure S6b (Supporting Information), the XAS spectrum of the cycled cathode closely resembles that of the pristine cathode, suggesting that if Mn_3_O_4_ is indeed formed, its quantity is relatively minor compared to the bulk of the cathode material.

Our findings are consistent with the dissolution/redeposition mechanism proposed in the literature.^[^ ^5^ ^]^ Such findings provide direct chemical evidence of a passivating Mn‐contained layer, which has been implicated as a cause of increasing overpotential. The advantage here is that XAS directly identifies the valence of Mn in the layer (Mn^2^⁺ → Mn^3^⁺) rather than inferring it indirectly. While the XANES fingerprint strongly points to the hydroxide form of Mn, additional analyses, such as in situ X‐ray diffraction or X‐ray photoelectron spectroscopy, could complement these findings to pin down the exact phase of the surface layer. Nevertheless, Mn valence changes captured by GI‐XAS are unambiguous. This experiment demonstrates how in situ GI‐XAS can detect subtle interfacial transformations that bulk measurements or electrochemical data alone would miss. By applying our depth‐sensitive probe, we were able to link an observed electrochemical symptom (temporary voltage rise and capacity loss) to its mechanistic origin: surface accumulation of Mn^2^⁺ species and incomplete reoxidation. Such an insight is valuable for devising mitigation strategies. For example, adding a Mn^2^⁺ chelating additive to the electrolyte might suppress Mn^2^⁺ dissolution, or a protective coating on the MnO_2_ could prevent direct contact with the electrolyte, thereby avoiding the MnO_2_ → Mn^2^⁺ reaction. These strategies could help maintain the integrity of the cathode surface and improve long‐term performance.

## 4 Conclusion

This work highlights the capability of synchrotron X‐ray techniques to monitor surface‐specific chemical changes during the operation of the MnO_2_ cathode in ZIBs. By tuning the X‐ray incidence angle, we were able to separately examine the bulk and the surface of the MnO_2_ cathode in the in situ condition. Besides, GI‐XAS data revealed that while the bulk of MnO_2_ undergoes reversible Zn^2^⁺ insertion/extraction, with Mn valence cycling between +4 and +3, the cathode‐electrolyte interface experiences additional processes. Thus, Mn dissolves as Mn^2^⁺ into the electrolyte at deep discharge and reprecipitates as a Mn hydroxide‐like layer on charge, which only partly reverts to MnO_2_. This interfacial Mn loss and deposition process provides a mechanistic explanation for the capacity fading and increased overpotential observed in Zn‐MnO_2_ systems. Such insights were not accessible via conventional in situ techniques that probe the whole electrode without depth resolution. The novelty of in situ GI‐XAS lies in its ability to focus on the solid–liquid interface, which is where many failure mechanisms in batteries originate. We believe that continued development of in situ surface‐sensitive X‐ray techniques, such as GI‐XAS, total reflection XAS, or related methods, will play a key role in unraveling the complex interphase phenomena in next‐generation batteries. Ultimately, based on the formation extracted by this characterization, more durable and high‐performance energy storage devices can be designed.

## Conflict of Interest

The authors declare no conflict of interest.

## Supporting information



Supporting Information

## Data Availability

The data that support the findings of this study are available from the corresponding author upon reasonable request.
