# Supplementary material for: Monitoring Interfacial Dynamics of a Zinc‐Ion Battery Cathode Using In Situ Grazing Incidence X‐Ray Absorption Spectroscopy: A Case Study of Manganese Dioxide
Source: Small Methods. 2025 Aug 5;10(3):e00871. doi: 10.1002/smtd.202500871 (PMC12893264; doi:10.1002/smtd.202500871)
Supplement: Supplementary file 1 — Supporting Information [file SMTD-10-e00871-s001.docx]

**Monitoring Interfacial Dynamics of a Zinc-Ion Battery Cathode using In-situ Grazing Incidence X‑ray Absorption Spectroscopy: A Case Study of Manganese Dioxide**

Wathanyu Kao-ian^1^, Phonnapha Tangthuam^1^, Pinit Kidkhunthod^2^, Wanwisa Limphirat^2^, Jintara Padchasri^2^, Nicolas Aubert^3^, Gianluca Ciatto^3^, Insik In^1,4,5,*^, Kevin C.-W. Wu^1,6,7^, Soorathep Kheawhom^1,8,*^

^1^ Department of Chemical Engineering, Faculty of Engineering, Chulalongkorn University, Bangkok 10330, Thailand

^2^ Synchrotron Light Research Institute (Public Organization), 111 University Avenue, Muang District, Nakhon Ratchasima 30000, Thailand

^3^ Synchrotron SOLEIL, L'Orme des Merisiers, Départementale 128, 91190 Saint-Aubin, France

^4^ Department of Polymer Science and Engineering, Chemical Industry Institute, Korea National University of Transportation, Chungju, 27469, South Korea

^5^ Department of IT-Energy Convergence (BK21 FOUR), Korea National University of Transportation, Chungju, 27469, South Korea

^6^ Department of Chemical Engineering, National Taiwan University, Taipei, 10617, Taiwan

^7^ Department of Chemical Engineering and Materials Science, Yuan Ze University, Chung-Li, Taoyuan, Taiwan

^8^ Center of Excellence on Advanced Materials for Energy Storage, Chulalongkorn University, Bangkok 10330, Thailand

*Corresponding author: in1@ut.ac.kr (Insik In), Soorathep.k@chula.ac.th (Soorathep Kheawhom)

**Supporting information (SI)**


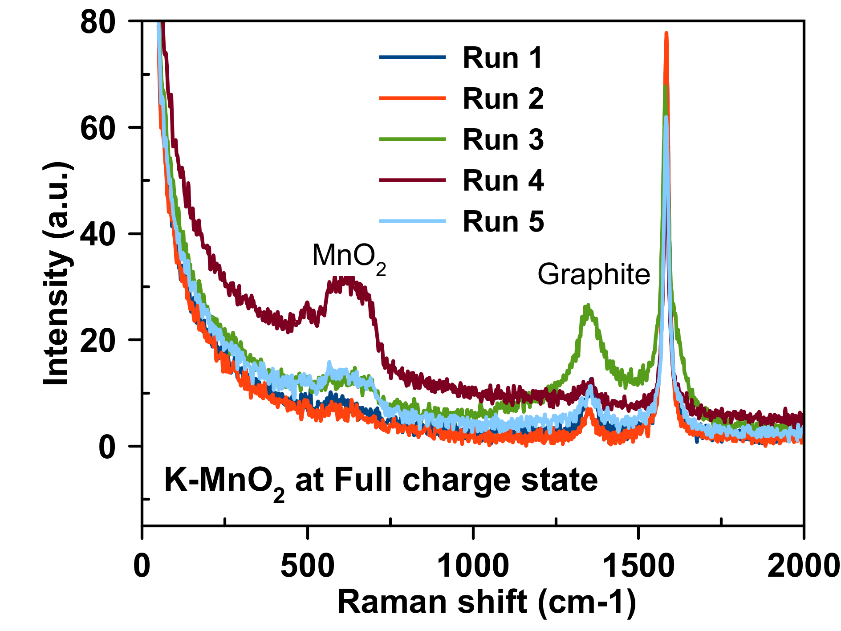


**Fig. S1.** Raman spectra of the same K-MnO₂ cathode collected from different runs.


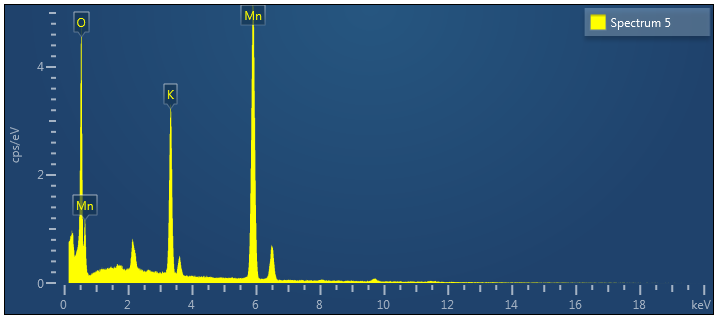


**Fig. S2.** EDS spectrum of the K-MnO_2_ powder.

**Table S1.** Fitting result of the EDS spectrum of Fig. S1.

| **Spectrum 5** |  |  |  |  |
| --- | --- | --- | --- | --- |
| **Element** | **Line Type** | **Weight %** | **Weight % Sigma** | **Atomic %** |
| O | K series | 31.86 | 0.31 | 59.95 |
| K | K series | 12.18 | 0.12 | 9.38 |
| Mn | K series | 55.96 | 0.28 | 30.67 |
| Total |  | 100.00 |  | 100.00 |


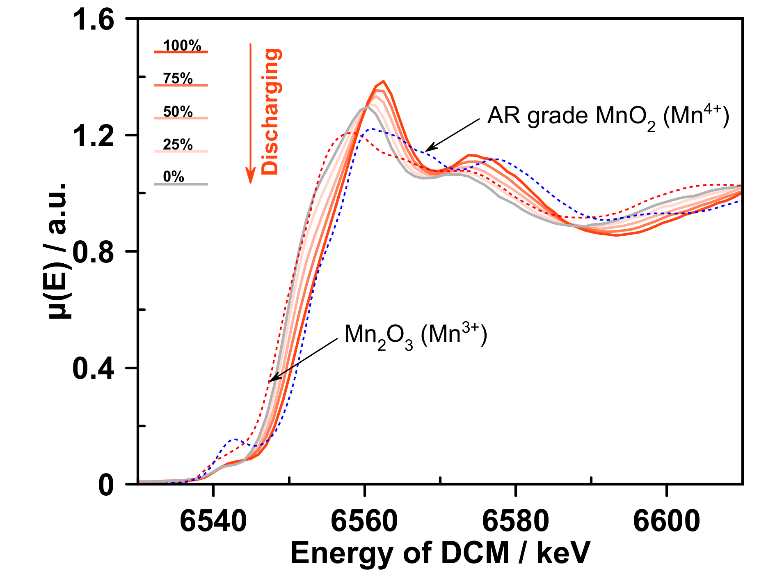


**Fig. S3.** In-situ XANES spectra of the Zn/MnO_2_ battery obtained using transmission mode XAS, reproduced from reference^[1]^.

**Preparation of electrolyte samples for Mn dissolution analysis**

Inductively Coupled Plasma Optical Emission Spectroscopy (ICP-OES) was employed to verify the dissolution of Mn species during the discharge process of the MnO₂ cathode, particularly near the fully discharged state. The electrolyte samples were obtained from cells assembled in a pouch cell configuration. Each pouch cell contained a cathode (7.5 × 6.2 cm²) with 130 mg of MnO₂ and 15 mL of 0.5 M Zn(OTf)₂ in dimethylformamide (DMF) as the electrolyte. One cell was discharged to full capacity, while another was discharged and then recharged to approximately half of its original capacity. After cycling, the electrolyte from each cell was extracted and filtered. Current density used in charging/discharging was ~ The resulting liquid samples were then analyzed using the ICP-OES technique. Result is as shown in Fig. S4. The results indicate the presence of Mn in the electrolyte at the discharged state, with its concentration decreasing upon charging.


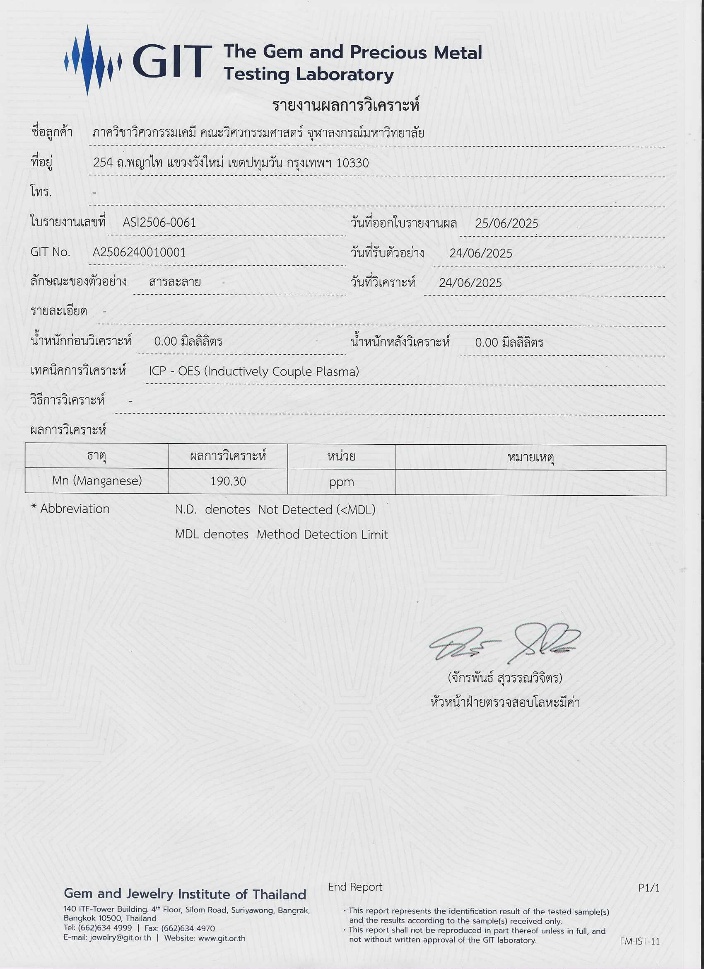

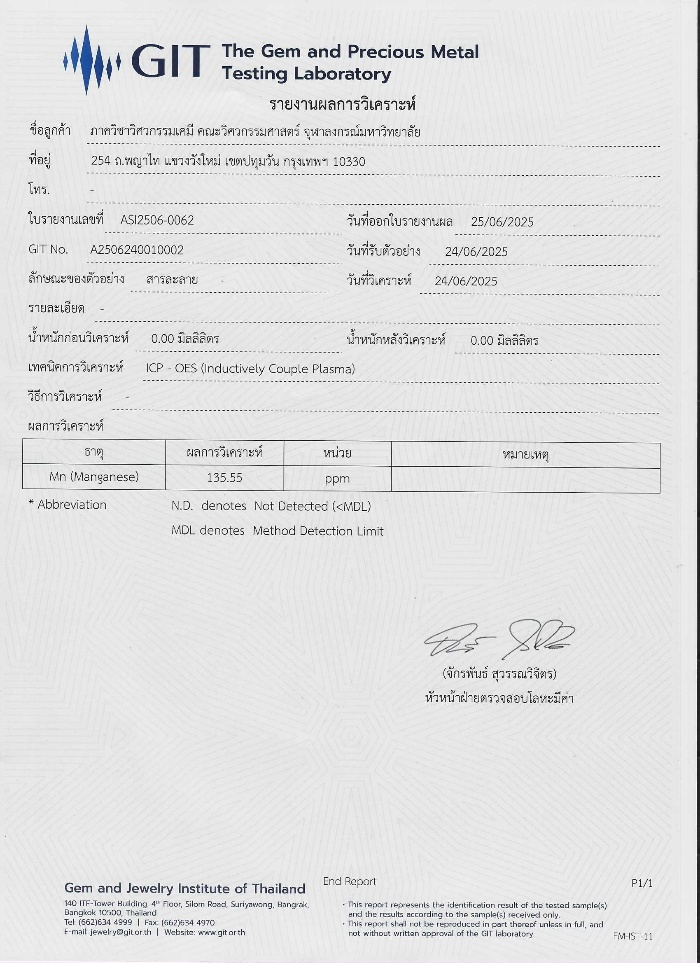


**(b)**

**(a)**

**Fig. S4.** Certification of ICP-OES result that confirm the existence of Mn specie in electrolyte during: (a) full-discharge (~190 ppm) and (b) after half-charging (~135 ppm).

**Preparation of coin cell for galvanostatic cycling stability test and electrochemical impedance spectroscopy (EIS) technique**

The cathode sheet and 50 µm Zn foil were punched into 16 mm diameter disks using an electrode puncher. Glass microfiber filters (Whatman GF/A) were cut into 19 mm disks and used as separators. CR2025 coin cell components were employed for cell assembly, including a 0.5 mm spacer, 1.3 mm cone spring, negative electrode cap, and positive electrode cap. The cells were sealed using a coin cell crimper. Galvanostatic charge–discharge tests were performed with an initial current density of 50 mA/g based on the MnO₂ loading, using a voltage window of 0.6–1.85 V for the first three cycles. The tests were conducted using a NEWARE BTS4000 battery tester. Result is shown in Fig. 4e. Subsequently, the current density was increased to 200 mA/g for cycling up to 100 cycles. Electrochemical impedance spectroscopy (EIS) measurements were performed at the 6th, 25th, and 100th cycles using a Biologic SP-50e instrument.

In Fig. 4f, all spectra exhibit two distinct semicircles, corresponding to the electrochemical characteristics of the anode and cathode. In most zinc-ion battery studies^[2]^, the high-frequency semicircle is typically attributed to the anode, while the low-frequency semicircle represents the cathode. However, definitive assignment requires further validation. Therefore, additional EIS measurements were performed at different states of charge, namely, the fully charged and fully discharged states. Given that the cathode is the rate-limiting component in the cell, which governs the discharge capacity, its charge-transfer resistance is expected to increase significantly in the discharged state. As shown in Fig. S5, the second semicircle (2) from the left, corresponding to the cathode, is substantially larger in the discharged state compared to the charged state. This observation supports the assignment of the first semicircle (1) to the Zn anode and the second semicircle (2) to the cathode.

To evaluate the evolution of the cathode’s charge-transfer resistance during cycling, EIS data were fitted using Zsimpwin software (version 3.60). The equivalent circuit model LR(QR)(QR)W was applied for the fitting.


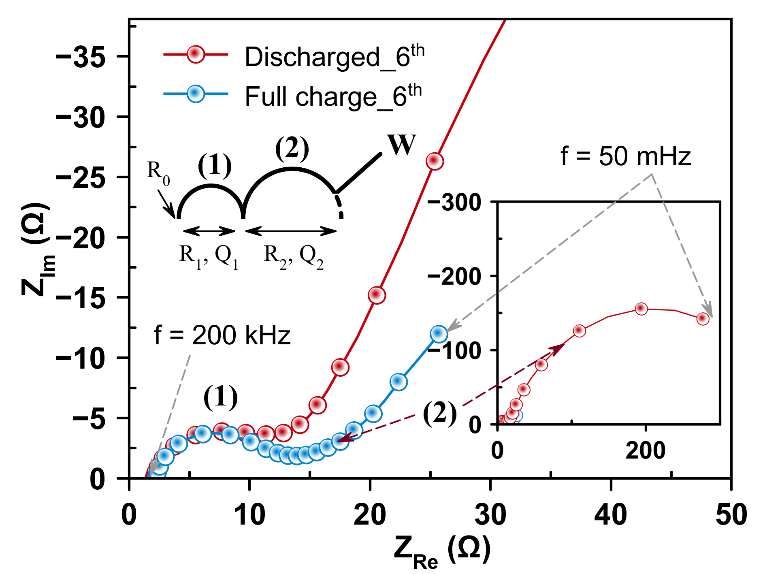


**Fig. S5.** EIS spectra of: full charge and discharged battery.

**Table S2.** Fitted result of EIS data from Fig. 4f using model: LR(QR)(QR)W (Zsimpwin).

| **Cycle number** | **L (10^7^H)** | **R_0_ (Ω)** | **R_1_ (Ω)** | **Q_1_** | **R_2_ (Ω)** | **Q_2_** | **10^2^W** |
| --- | --- | --- | --- | --- | --- | --- | --- |
| 6^th^ | 1.548 | 2.161 | 7.933 | 10^5^Y0: 3.739  n: 0.873 | 5.189 | 10^3^Y0: 7.023  n: 0.566 | 11.21 |
| 25^th^ | 1.592 | 1.920 | 11.50 | 10^5^Y0: 4.614  n: 0.864 | 10.90 | 10^3^Y0: 7.475  n: 0.553 | 11.79 |
| 100^th^ | 1.826 | 1.946 | 24.91 | 10^5^Y0: 3.611  n: 0.882 | 50.25 | 10^3^Y0: 4.439  n: 0.579 | 2.777 |


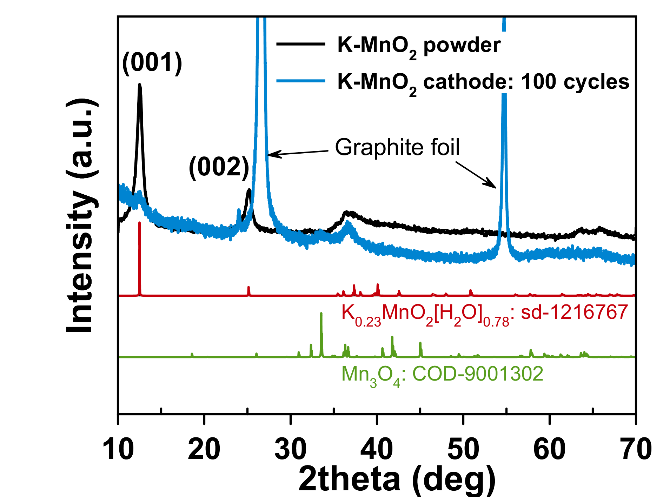

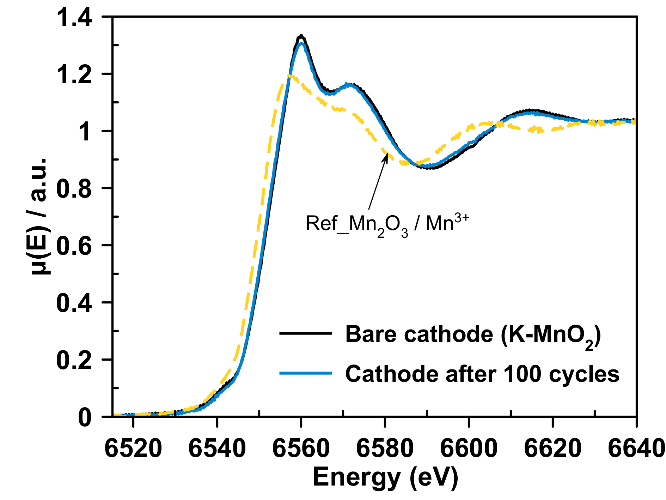


**(b)**

**(a)**

**Fig. S6.** (a) XRD patterns of the pristine K-MnO₂ powder and the cathode after 100 charge–discharge cycles. (b) XAS spectra of the pristine K-MnO₂ cathode and the cycled cathode after 100 cycles: data was collected at beamline2.2, SLRI^[3]^.

**Reference**

[1] W. Kao-ian, J. Sangsawang, M. Gopalakrishnan, S. Wannapaiboon, A. Watwiangkham, S. Jungsuttiwong, J. Theerthagiri, M. Y. Choi, S. Kheawhom, *ACS Appl. Mater. Interfaces* **2024**, *16*, 56926.

[2] J. Xie, Z. Jia, H.-S. Tsai, M. Qian, X. Zhang, *Chem. Eng. J.* **2025**, *518*, 164660.

[3] P. Villars, K. Cenzual, “K-birnessite (K_0.23_MnO_2_[H_2_O]_0.78_ tricl) Crystal Structure: Datasheet,” can be found under https://materials.springer.com/isp/crystallographic/docs/sd_1216767, **2025**.
